# The Prostate Care Questionnaire for Carers (PCQ-C): reliability, validity and acceptability

**DOI:** 10.1186/1472-6963-9-229

**Published:** 2009-12-11

**Authors:** Paul Sinfield, Richard Baker, Carolyn Tarrant, Shona Agarwal, Andrew M Colman, William Steward, Roger Kockelbergh, John K Mellon

**Affiliations:** 1 Department of Health Sciences, University of Leicester, Leicester, UK; 2 School of Psychology, University of Leicester, Leicester, UK; 3 Department of Cancer Studies & Molecular Medicine, University of Leicester, Leicester, UK

## Abstract

**Background:**

Patient experience is commonly monitored in evaluating and improving health care, but the experience of carers (partners/relatives/friends) is rarely monitored even though the role of carers can often be substantial. For carers to fulfil their role it is necessary to address their needs. This paper describes an evaluation of the reliability, validity and acceptability of the PCQ-C, a newly developed instrument designed to measure the experiences of carers of men with prostate cancer.

**Methods:**

The reliability, acceptability and validity of the PCQ-C were tested through a postal survey and interviews with carers. The PCQ-C was posted to 1087 prostate cancer patients and patients were asked to pass the questionnaire on to their carer. Non-responders received one reminder. To assess test-retest reliability, 210 carers who had responded to the questionnaire were resent it a second time three weeks later. A subsample of nine carers from patients attending one hospital took part in qualitative interviews to assess validity and acceptability of the PCQ-C. Acceptability to service providers was evaluated based on four hospitals' experiences of running a survey using the PCQ-C.

**Results:**

Questionnaires were returned by 514 carers (47.3%), and the majority of questions showed less than 10% missing data. Across the sections of the questionnaire internal consistency was high (Cronbach's alpha ranging from 0.80 to 0.89), and test-retest stability showed moderate to high stability (intraclass correlation coefficients ranging from 0.52 to 0.83). Interviews of carers indicated that the PCQ-C was valid and acceptable. Feedback from hospitals indicated that they found the questionnaire useful, and highlighted important considerations for its future use as part of quality improvement initiatives.

**Conclusions:**

The PCQ-C has been found to be acceptable to carers and service providers having been used successfully in hospitals in England. It is ready for use to measure the aspects of care that need to be addressed to improve the quality of prostate cancer care, and for research.

## Background

In England, a major programme of investment and reform in cancer care was set out in the NHS Cancer Plan 2000 [1]. A national patient survey [2] found signs of progress in terms of patient experience, but patients with prostate cancer tended to report smaller improvements than other cancer patients. The reasons for this were not understood and this led the authors of the report to recommend that cancer networks should give particular attention to prostate cancer [3]. Monitoring patients' experience of care is essential to understand and improve the delivery of care [4] but no standardised measure for prostate cancer patients was available. Consequently, the NHS Service Delivery and Organisation in association with the national clinical director for cancer commissioned the development of a measure of the experiences of care of men with prostate cancer. The measure was for use in routine practice to compare performance, assess the impact of innovations, and to direct resources more appropriately.

We interpreted carers' experience of prostate cancer care to relate to carers' reports of specific aspects of care, including their experience of being provided with information and advice, involvement in care and decision making, provision of support, delays in care, and coordination of care. Thus carer experience focuses on the process of care, and is conceptually distinct from concepts such as satisfaction (a complex outcome incorporating expectations, experience, feelings, and importance of aspects of care), and quality of life (the impact of physical and mental health on functioning). Furthermore, the findings from measures of experience are usually are regarded as easier to put into practice than measures of satisfaction [5].

The research undertaken to inform this measure established both the importance of the role of carers (partners/relatives/friends) of men with prostate cancer and the need for a separate measure of their experience of care that would enable their needs to be addressed. While patient experience (or satisfaction) is commonly monitored in evaluating and improving health care, the experience of carers is monitored more rarely and is usually focused on their views of the care that the patient receives (e.g. palliative care). However, this approach does not consider the experiences or needs of carers themselves who may experience significant distress [6] and may need help to support the patient [7]. In order to enable carers to cope with their anxieties and fulfil their supportive role, it is important to understand their own experiences during the health care of their partner/relative/friend for prostate cancer.

This paper describes the development of a questionnaire to measure the experiences of carers of men with prostate cancer- the Prostate Care Questionnaire for Carers (PCQ-C). Measures of experience suitable for wide use must be developed systematically to ensure they address the issues that are important to patients or carers, are readily understood, and are reliable and valid. They must also meet the needs of providers so that the measures can be used flexibly and at different phases of care depending on the providers' focus for quality improvement, and be administered and analysed efficiently. Our aim was to develop a valid, reliable and usable measure that can be used in routine practice as well as in studies of new ways of delivering care. This paper reports the formal evaluation of the reliability and validity of the complete PCQ-C, as well as assessments of its acceptability to carers and service providers. A companion paper describes the development of the measure for use with men with prostate cancer (PCQ-P) [8].

## Methods

### Development and characteristics of the PCQ-C

The PCQ-C (Prostate Care Questionnaire for Carers) was developed in response to preliminary research with healthcare professionals that identified a need for a separate measure of carers' experience of care. The format and content of the measure were identified through a literature review [9] and, interviews with carers [10] and service providers from the NHS and the voluntary sector [11]. This ensured the involvement of both those with experience of the carer role in prostate cancer and experience of administering measures to service users. The carer measure developed was a questionnaire designed to cover the issues which included: information and explanations, the opportunity to be involved in consultations, provision of practical caring advice and where to find sources of support. The questionnaire is divided into four sections: carer experiences when the patient is undergoing testing for prostate cancer (Section A, 13 questions); carer experiences during getting the diagnosis and making the treatment decision (Section B, 26 questions); carer experiences during treatment, discharge and monitoring (Section C, 18 questions); health and socio-demographic questions (Section D, 7 questions). Sections may be administered in appropriate combinations to cover a phase, or phases of care, recently experienced and to keep the number of questions to be answered by carers to a manageable number. The questionnaire was subject to thorough piloting in three hospitals [11] and, along with the user guide, is available online for download and use [12]. A short version of the questionnaire (PCQ-Cs, 18 questions) has also been developed to cover the issues most important to carers [12].

### Measures of reliability, validity and acceptability to carers

Five hospitals in England were selected to participate in the study to test reliability, validity and acceptability, representing a range in terms of urban and rural locality, teaching and non-teaching hospitals and foundation trust status. Each hospital drew a consecutive sample of all patients who had been diagnosed with, or treated for, prostate cancer within the past two years, excluding patients who had died or were too ill to participate; this produced a list of between 152 and 253 patients per hospital. Details of the participating hospitals and patients are given in the accompanying paper [8]. All 1087 patients who had been invited to participate in the testing of the PCQ-P (Prostate Care Questionnaire for Patients) were also mailed a copy of the PCQ-C. Patients were asked to pass on a pack containing the PCQ-C (either Sections A, B and D, Sections C and D or the complete questionnaire) to their carer if they were able to identify a person as their carer, and were happy to pass on the questionnaire. It was recognised that many people caring for a relative or friend with prostate cancer may not identify with the designation 'carer', so the terms 'partner/relative/friend' were used alongside the term 'carer'. The pack carers received contained the carer questionnaire, a covering letter, and an information sheet, and carers were asked to return their questionnaire in the separate stamped-addressed envelope provided.

Test-retest reliability was undertaken in two hospital sites (hospitals 1 and 2). In these sites, 210 carers who had responded to the original mailing were posted the PCQ-C again three weeks later. In addition, nine carers from one hospital site who had completed the PCQ-C took part in semi-structured interviews to explore acceptability and face validity. Four face-to-face and five telephone interviews were carried out. Interviews were not transcribed, but notes were taken during the interview by the interviewer.

Key properties of the sections of the PCQ-C, including aspects of validity, reliability, and acceptability to carers were analysed. Overall scores were calculated for each section of the questionnaire, by summing up scores across questions and converting to a score out of 100, with higher scores indicating more positive experiences of care [11]. Statistical analyses were conducted using SPSS version 16.0.

#### Acceptability to carers

Acceptability was evaluated through examination of completion rates for individual questions and whole sections of the questionnaire, and by analysing distributions of responses for individual questions. Acceptability was also assessed in interviews, by asking carers about their experience of completing the questionnaire.

#### Validity

Face and content validity were investigated through the carer interviews. Carers were asked to recount their experiences of the care process and their involvement in it, and to reflect on their responses to the questionnaire. Carers were also asked whether there were any issues that were important to them that were not included in the PCQ-C. Content validity was also assessed through exploratory principal components analysis (PCA), to identify if key aspects of care identified in the preliminary research [9,10] had been incorporated into the PCQ-C.

In the absence of a comparable carer questionnaire further validity testing was undertaken by comparing the scores for patients and carers in corresponding sections of their respective questionnaires. A moderate to high correlation (0.3) between patient and carer scores was expected, reflecting differences in the questions included in the patient and carer questionnaire, but also reflecting the fact that patients and carers may have different perspectives on the same experience.

#### Reliability

Internal consistency reliability for each section was measured using Cronbach's alpha [13]. Stability reliability was assessed using intraclass correlation coefficients (ICC) between scores on the first and second completion of the questionnaire. Stability was also assessed by calculating the percentages of carers answering each question in the same way on the first and second completion of the questionnaire.

#### Usability and acceptability to service providers

The usability and acceptability of the questionnaire to service providers was assessed through a sample of four hospitals surveying carers' experiences using different sections of the PCQ-C, and then seeking feedback on their experiences of this process. Four hospitals in England were selected to ensure a range in terms of urban and rural locality, teaching and non-teaching hospitals, and foundation trust status. Hospitals were provided with questionnaires, a user guide developed as part of the study, and software to enable them to enter their data and produce basic summary results. Hospitals were asked to survey around 100 carers (via the patients) and feedback on staff's experiences was gained through semi-structured interviews with one or two key persons who had administered the survey in each hospital (total of 5 interviews), along with informal discussion with other members of hospital staff. Interviews were not transcribed, but notes were taken by the interviewer during the interview.

## Results

### Acceptability to carers

Questionnaires were returned by 514 carers. As the carer questionnaires were sent to 1087 patients this equates to a response rate of 47.3%. However, the number of patients who did not have a carer or who chose not to pass the invitation pack to their carer is unknown, meaning that the true response rate cannot be calculated. Sections A and B were completed by 244 carers, and 350 completed Section C. The demographic characteristics and health status of responders are summarised in Table 1.

**Table 1:** Demographic characteristics and overall health status of carer sample: reliability and validity testing

Relationship to patient	*N *(%)^i^
Wife/partner	444 (86.4)
Other relative	13 (2.2)
Friend	2 (0.3)
Other	22 (3.7)

**Age (years)**	
< 54	68 (11.4)
55-64	190 (31.9)
65-74	225 (37.8)
75+	96 (16.1)

**Overall health**	
Very good	154 (25.9)
Good	280 (47.1)
Fair	118 (19.8)
Poor	18 (3.0)
Very poor	5 (0.8)

**Ethnicity**	
White British/Irish	562 (94.5)
South Asian	3 (0.5)
African/Caribbean	5 (0.8)
Other	0 (0)

**Current situation**	
Employed	136 (22.9)
Retired	388 (65.2)
Other	35 (5.9)

**Current or most recent occupation**	
Professional	136 (22.9)
Managerial	51 (8.6)
Clerical	164 (27.6)
Technical/craft	7 (1.2)
Manual/service	82 (13.8)

^i^May not add to 100 due to missing data

The proportion of carers completing fewer than 50% of the questions in each section was low to moderate: 52 carers (15.9%) for Section A, 28 carers (13.5%) for Section B, and 62 (27.1%) for Section C. Missing data were usually due to carers omitting whole sections, and in many cases this may have been appropriate. For example, carers who indicated that the patient was not undergoing active treatment were more likely to leave Section C (experiences during treatment, discharge and monitoring) blank (χ^2 ^= 38.3, p < 0.001). For carers who completed more than 50% of the questionnaire, missing data for individual questions ranged from 0% - 32.4%. However, the majority of questions showed less than 10% missing data. Responses to most questions were well distributed across response options. Overall, carers more often reported positive experiences, with mean overall scores ranging from 57.0 to 86.0 across the sections. Descriptive statistics of section scores are shown in Table 2.

**Table 2:** Descriptive statistics of overall scores from the three sections of the questionnaire

Section^i^	*N*	Mean score	*SD*	Minimum	Maximum	% with lowest possible score	% with highest possible score
Section A	179	57.0	27.7	4.7	100	0	3.7
Section B	167	86.0	16.6	16.7	100	0	13.1
Section C	278	69.2	27.0	0	100	0.9	14.3

^i^Section A = carer experiences when the patient was undergoing testing for prostate cancer, Section B = carer experiences during getting the diagnosis and making the treatment decision, Section C = carer experiences during treatment, discharge and monitoring

### Face and content validity

All carers interviewed agreed that the questionnaires covered important aspects of care. Several questions were felt to be ambiguous and the wording was changed to improve understanding. One issue, the importance of a well organised discharge, was highlighted by several carers, who reported having experienced significant problems at this phase of care. Consequently, two further questions on the provision of information about recovery and side-effects, and obtaining medical supplies were added to the final version of the questionnaire.

Content validity was also assessed through exploratory principal components analysis. Section A (tests) emerged as a single component, including all questions. In Section B (diagnosis and treatment decision) and Section C (treatment and monitoring) three components emerged. On inspection the components in Section B were related to 'involvement and information', 'explanation', and 'treatment decision'. In Section C the components related to 'explanation, information and support', 'discharge', and 'continuity and communication'. Comparison of these components with themes from the initial carer interviews [10] and literature review [9] confirmed that key aspects of care identified in this preliminary research had been incorporated into the PCQ-C (see additional file 1: exploratory PCA).

The comparison of patient and carer scores for corresponding sections of their respective questionnaires showed moderate to high, significant, correlations (see Table 3) providing support for the validity of the carer questionnaire.

**Table 3:** Correlation between carer and patient scores

Carer and Patient Sections	Correlation: Pearson's r *P value* N
Carer Section A (referral and tests) with Patient Section B (tests at the hospital)	**0.43** *p < 0.001* 169
Carer Section B (diagnosis) with Patient Section C (diagnosis)	**0.62** *p < 0.001* 148
Carer Section C (treatment and monitoring) with Patient Section D (treatment)	0.59 *p < 0.001* 199

### Internal consistency reliability

Cronbach's alpha coefficients ranged from 0.80 to 0.89, indicating high internal consistency for all sections of the PCQ-C (see Table 3).

### Test-retest reliability

Ninety-two carers (43.8%) completed the retest questionnaire; 43/88 (48.9%) completed Sections A and B, and 49/122 (40.2%) completed Section C. Carers completing retest questionnaires did not differ significantly from other carers in terms of age, health status, ethnic group, or employment status (*p *> 0.05 in each case). The test-retest intraclass correlation coefficients for the three sections were between 0.52 and 0.83, and all were significant at *p *< 0.05 (see Table 4), indicating acceptable reliability [14]. The stability of section B is somewhat lower than the other sections. The consistency of responses to individual questions was high, between 58.3% and 100% of carers answering identically on the first and second mailing. Most questions (52, 89.7%) were answered perfectly consistently by over 70% of responders on the first and second completion of the questionnaire. The questions where responses were less consistent were those with a higher number of response options. For these questions, the difference between carer responses on the first and second completion of the PCQ-C tended to be a shift to the neighbouring response option, for example, from 'good' to 'very good'.

**Table 4:** Reliability: Internal consistency and stability of the three sections of the PCQ-C

	Internal consistency	Stability: Test-retest reliability		
**Section^i^**	**Cronbach's α**	**1st mailing mean score** ***SD*, min-max** **(*N*)**	**2nd mailing mean score** ***SD*, min-max** **(*N*)**	**Intraclass Correlation Coefficient (ICC)**
Section A	0.80	65.4 26.7, 14.8-100 (29)	63.9 23.2, 12.7-96.5 (28)	0.77
Section B	0.82	90.2 16.1, 16.7-100 (29)	94.0 7.5, 72.2-100 (27)	0.52
Section C	0.89	80.7 20.9, 9.1-100 (40)	77.9 23.9, 8.3-100 (43)	0.83

^i^Section A = carer experiences when the patient was undergoing testing for prostate cancer, Section B = carer experiences during getting the diagnosis and making the treatment decision, Section C = carer experiences during treatment, discharge and monitoring

### Sensitivity

The results from the hospitals where PCQ-C was used show that there are variations in reported experiences of care between hospitals as well as experience of some aspects of care that is poor, such as tests and monitoring (see Table 5).

**Table 5:** Summary scores by hospital - PCQ-C

		N	Mean	Std. Deviation	F value for difference between means for individual hospitals (p value)
Score for Section A	Hospital 1	74	61.0^a^	27.3	3.31 *p = 0.04*
	Hospital 3	48	48.4^a^	26.5	
	Hospital 5	57	59.0	28.0	
	*Total*	*179*	*57.0*	*27.7*	
Score for Section B	Hospital 1	67	89.6^a^	14.5	3.83 *p = 0.02*
	Hospital 3	47	80.9^a^	21.9	
	Hospital 5	53	86.0	12.1	
	*Total*	*167*	*86.0*	*16.6*	
Score for Section C	Hospital 2	112	77.8^a, b^	23.5	10.55 *p < 0.001*
	Hospital 4	117	62.2^a^	28.7	
	Hospital 5	49	66.3^b^	25.5	
	*Total*	*278*	*69.2*	*27.0*	

^a, b ^Means with the same superscript differ significantly from each other at p < 0.05. Other differences are not significant

### Acceptability to service providers

No major difficulties were reported by the four hospitals administering the questionnaire (all conducted a postal survey and one supplemented this by handing questionnaires out in the Urology clinic). In interviews with hospital staff the importance of introducing the measure sensitively was emphasised, as was the need to be clear about how the results would be used, as this would help to ensure staff co-operation. Their feedback on the questionnaire itself was that it was relevant and would provide useful data, allowing comparison between hospitals as well as identifying specific aspects of service delivery that should be examined.

## Discussion

In England, men's reported experiences of prostate cancer care has been worse than for patients with other cancers, and since a high proportion of patients and their carers are relatively old, deficiencies in care are likely to have a significant impact on both patients and carers. However, there are no measures specifically designed to address the experiences of these carers and that could be used to monitor their needs. This paper has explained the development of the measure for carers, PCQ-C, which has been designed so that it can be used independently or alongside the measure of patients' experience of prostate cancer care, PCQ-P [8]. Corresponding sections from each questionnaire can be administered simultaneously to carers and patients respectively.

The strengths of the PCQ-C are that the form and content have been based on research with users and healthcare professionals (clinical and administrative staff). The questionnaire has been subjected to a range of tests for validity and reliability in a variety of hospital settings, so that it is ready for use in hospitals. The PCQ-C can be used as a complete questionnaire, or sections of the measure can be used individually. A short version, covering carer experiences across the whole patient journey, is also available [11]. Feedback from four hospitals on their experience of using the PCQ-P and PCQ-C indicated that they found the questionnaires relevant and easy to use, and felt that they produced useful data [8]. Further use of the questionnaire could be to review and compare care between hospitals in a region, or to establish national benchmarks for care against which all hospitals can compare themselves. Ideally the use of the questionnaire should be accompanied by efforts to tailor service delivery to the needs of carers.

There are several limitations that should be noted. It was not possible to test for criterion validity because there was no accepted, valid carer experience questionnaire available with which PCQ-C could be compared. The stability of section B is somewhat lower than for the other sections. The majority of the carers who completed Section B on both the first and the second occasion gave high overall scores for the section, and lack of variance in scores for Section B may account for the lower ICC. In addition sample size for the analysis of carer data is relatively small. There remain significant barriers to accessing carers, not least the requirement to invite carers to participate in studies via patients, and some consideration of how best to recruit carers when using the PCQ-C is required. Opportunistic sampling (e.g. via support groups and charities) may provide a convenient alternative route to the approach used in this study, although this will carry an increased risk of bias [15].

There are several opportunities for further research. Firstly, this study covered experiences of prostate cancer care from initial consultation through to treatment and monitoring. To cover all the phases of care a measure is needed that addresses experiences during palliative and end-of-life care. Secondly, to further understanding of carers and their experiences comparisons between groups of carers may be carried out using the existing measure (e.g. looking at responses and socio-demographic characteristics). Thirdly, while the questionnaire was able to detect different experiences of carers between different hospitals its sensitivity to change has yet to be tested. This could be carried out for example by using PCQ-C to measure carer experience before and after a change to service delivery.

There are two aspects of the PCQ-C that are worth noting. Firstly, it is a questionnaire that has been tested and exhibits satisfactory reliability, validity and acceptability to carers of men with prostate cancer. The PCQ-C is ready to be used in service monitoring and improvement, and could also provide a starting point for the development of instruments for other cancer groups, as many of the issues that it covers are relevant to other cancers. Secondly, the questionnaire has been developed as a measure of carers' own experience of prostate cancer care so that service delivery can be tailored to meet carers' needs, and enable them to be more effective in providing patient support. This is in contrast to other measures that address carers' experience of, or satisfaction with, the care that the patient has received (e.g. palliative care), or that measure emotional wellbeing or strain on carers [16]. The PCQ-C could also be used to identify the impact of positive and negative experiences of service provision on carer well-being. Further research using the PCQ-C could also identify what changes and interventions improve carers' experiences. This would allow service delivery to be tailored in such as way as to reduce the strain experienced by carers. This represents a significantly different approach to the support of carers.

## Conclusions

The PCQ-C described here has the potential to provide valuable carer feedback to those working in health care throughout the different phases of care of prostate cancer patients, from testing to monitoring. These data can be used to tailor service delivery to improve carer experience, and equip them better to cope with providing support for the patient.

## Competing interests

The authors declare that they have no competing interests.

## Authors' contributions

RB was lead of the project and participated in the design of the study reported in this paper. PS, SA and CT participated in the data collection and analysis of the study reported in this paper, and PS prepared the first draft of this paper. All authors participated in the design and management of the study, and in reading and approving the final manuscript.

## Pre-publication history

The pre-publication history for this paper can be accessed here:

http://www.biomedcentral.com/1472-6963/9/229/prepub

## Supplementary Material

Additional file 1 **Exploratory Principal Components Analysis with Varimax rotation for each section of the PCQ-C**. Table showing results of an exploratory PCA for all sections of the PCQ-C. Click here for file
