# Supplementary material for: The Prostate Care Questionnaire for Carers (PCQ-C): reliability, validity and acceptability
Source: BMC Health Serv Res. 2009 Dec 11;9:229. doi: 10.1186/1472-6963-9-229 (PMC2797790; doi:10.1186/1472-6963-9-229)
Supplement: Additional file 1 — Exploratory Principal Components Analysis with Varimax rotation for each section of the PCQ-C. Table showing results of an exploratory PCA for all sections of the PCQ-C. [file 1472-6963-9-229-S1.DOCX]

Additional file 1: Exploratory Principal Components Analysis with Varimax rotation for each section of the PCQ–C^i^

**SECTION A: TESTS**

|  | **All Section A** |
| --- | --- |
| A11. Did the doctor or nurse at the hospital encourage you to go with him when he went back to the hospital for the diagnosis? | .783 |
| A9. Were YOU told beforehand: How long his test(s) would take / That he may be in pain/upset after the biopsy? | .727 |
| A10. Were you kept informed of where your partner/relative/friend was and how long he would be? | .711 |
| A4. Did the doctor recommend that it would be helpful for you to go with him to the hospital when he went for tests? | .687 |
| A12. Did a doctor or nurse offer you any support while you were waiting for the diagnosis? | .644 |
| A7. How would you rate the hospital facilities: Waiting area; Availability of refreshments; Toilets; Rooms where the tests were carried out? | .581 |
| A13. Have staff in different places worked well together when caring for your partner/relative/friend for this condition? | .560 |
| A3. Did the GP/nurse give your partner/relative/friend any written information about his tests? | .525 |

**SECTION B: DIAGNOSIS AND TREATMENT DECISION**

|  | **Component 1: Involvement & Information** | **Component 2: Explanation** | **Component 3: Treatment decision** |
| --- | --- | --- | --- |
| B19. Did the doctor or nurse involve you as much as you wanted in the decision about which treatment to have? | .897 |  |  |
| B16. Did the doctor or nurse include you in the discussion on the treatment options and possible side effects? | .804 | .325 |  |
| B18. Did the doctor or nurse encourage you both to take your time before making a decision about which treatment to have? | .708 |  | .418 |
| B21. After the treatment decision had been made did the doctor or nurse tell you that you could discuss your partners’/relatives’/friends’ treatment decision again? | .648 |  |  |
| B22. Did the doctor or nurse give you information about who to contact for advice or support (e.g. specialist nurse, patient support group, charity)? | .517 | .392 |  |
| B17. Did the doctor or nurse offer you or your partner/relative/friend any written or printed information about: The treatment options; The side effects or consequences of the treatment options; What could be done about the side effects? | .377 |  |  |
| B12. Did the doctor or nurse clearly explain whether or not the cancer had spread outside the prostate? |  | .739 |  |
| B13. Did the doctor or nurse clearly explain the different treatment options? | .359 | .703 |  |
| B14. Did the doctor or nurse clearly explain the possible side effects or consequences of these treatment options? |  | .657 | .450 |
| B11. Did the doctor or nurse clearly explain how aggressive the cancer was likely to be? | .346 | .631 |  |
| B15. Did the doctor or nurse clearly explain what could be done about the side effects? |  | .488 | .375 |
| B7. Did the doctor or nurse clearly explain your partner’s/relative’s/ friend’s diagnosis? | .319 | .482 |  |
| B20. Were you confident that the treatment decision was the best one for your partner/relative/friend? |  |  | .710 |
| B10. How did you feel about the length of time between being given the diagnosis and discussing the treatment options? |  |  | .683 |
| B8. Did the doctor or nurse explain your partner’s/relative’s/friend’s diagnosis in a considerate way? |  |  | .509 |

**SECTION C: TREATMENT AND MONITORING**

|  | **Component 1:**  **Explanation, information, support** | **Component 2: Discharge** | **Component 3: Continuity and communication** |
| --- | --- | --- | --- |
| C8. Did the doctor or nurse give you any information about who to contact for advice or support (e.g. specialist nurse, patient support group)? | .805 |  |  |
| C5. Did the doctor or nurse clearly explain what was going to happen when he went for treatment? | .726 |  |  |
| C14. Did a doctor or nurse organise the aftercare services that you needed to help care for your partner/relative/friend (e.g. district nurse, health visitor, physiotherapist)? | .715 |  |  |
| C7. Were you kept up to date: With the progress of the treatment; With how well the treatment was going? | .701 |  | .305 |
| C16. Did the doctor or nurse tell you who to contact if you were worried about his condition after treatment at the GP’s practice or hospital? | .684 | .307 |  |
| C15. Did the doctor or nurse explain why there is a need for regular tests to check his condition (e.g. PSA blood test)? | .653 |  |  |
| C13. Were you or your partner given equipment or supplies (e.g. continence pads) to use at home to help you care for your partner? | .651 | .425 |  |
| C10. Did the doctor or nurse discuss with you how to cope with caring for him? |  | .888 |  |
| C11. Did the doctor or nurse give you enough information about caring for him? |  | .885 |  |
| C12. Did a doctor or nurse discuss with you how to manage any potential side effects of the treatment (e.g. continence, problems with sex, pain) |  | .847 |  |
| C9. Before he left the hospital or finished his treatment did the doctor or nurse explain to you what would happen next (e.g. arrangements for follow-up)? | .478 | .603 |  |
| C17. Have staff in different places worked well together when caring for your partner/relative/friend for this condition: Between GP’s practice and hospital; Between hospitals; Between different departments (e.g. Urology and Oncology)? |  |  | .802 |
| C3. Were you told that it might be helpful if you could go with him when he went for treatment e.g. by doctor or nurse in a letter from the hospital? | .332 |  | .692 |

^i^Number of components limited based on examination of eigenvalues and scree plots. Loadings of 0.3 and higher only shown
